# The damping and structural properties of dragonfly and damselfly wings during dynamic movement

**DOI:** 10.1038/s42003-021-02263-2

**Published:** 2021-06-15

**Authors:** Carina Lietz, Clemens F. Schaber, Stanislav N. Gorb, Hamed Rajabi

**Affiliations:** 1 Institute of Zoology, Functional Morphology and Biomechanics, Kiel University, Kiel, Germany; 2 Present Address: Division of Mechanical Engineering and Design, School of Engineering, London South Bank University, London, UK

**Keywords:** Biomechanics, Animal physiology

## Abstract

For flying insects, stability is essential to maintain the orientation and direction of motion in flight. Flight instability is caused by a variety of factors, such as intended abrupt flight manoeuvres and unwanted environmental disturbances. Although wings play a key role in insect flight stability, little is known about their oscillatory behaviour. Here we present the first systematic study of insect wing damping. We show that different wing regions have almost identical damping properties. The mean damping ratio of fresh wings is noticeably higher than that previously thought. Flight muscles and hemolymph have almost no ‘direct’ influence on the wing damping. In contrast, the involvement of the wing hinge can significantly increase damping. We also show that although desiccation reduces the wing damping ratio, rehydration leads to full recovery of damping properties after desiccation. Hence, we expect hemolymph to influence the wing damping indirectly, by continuously hydrating the wing system.

## Abstract (web summary)

Lietz, Rajabi and colleagues investigate the physiological and structural components of damping in insect wings. Notably, flight muscles and hemolyph appear to have no direct involvement in wing damping, compared to the function of the wing hinge.

## Introduction

Flying animals have evolved strategies to adjust many aspects of their flight performance, such as the flight speed, altitude, manoeuvrability, etc. Although these strategies are very diverse, they generally fall into two categories: (i) those that control wing motion and (ii) those that modulate wing shape^1–4^. Although wing motion is always controlled actively by flight muscles, the wing shape can be tuned by either or both active and passive mechanisms^5^.

In birds and bats, wing shape adjustments are mostly achieved by the active control of the wing shape using flight muscles^6,7^. Insect wings, however, lack muscles, except those situated in the thorax. Therefore, in contrast to birds and bats, the aerodynamic force generation in flying insects mainly relies on passive changes of the wing shape, and perhaps some minor shape controls by the thoracic muscles^8–12^.

A growing body of research on wing biomechanics gives an increasingly clear picture of the influence of the design and material properties of insect wings on their deformations under typical flight forces^13–22^. We know that wings consist of supporting and deformable regions^23,24^. While the supporting regions enable wings to withstand flight forces, the deformable regions provide wings with the deformability required for the shape change. The interactions between the two regions in flight yield a balance between stiffness and flexibility, thereby allowing beneficial wing deformations while preventing excessive bending^11^.

Although insect wings often experience accidental stresses, caused by wind gusts, predatory attacks and collisions with vegetation, very little is known about their response to the unexpected forces occurring under such circumstances^25^. Our current understanding is that insect wings are very resilient to environmental disturbances; they have evolved strategies to both reduce the risk of material/structural failures due to excessive loadings and isolate such failures, when they occur^25–30^. For instance, under an accidental collision, wings can reversibly bend without failure^27^, because some wing regions that do not typically deform in flight undergo large deformations under unexpected loads^13^. And, if a defect occurs, veins prevent its propagation by working as mechanical barriers ahead of the growing defect^29–32^. What remains fully unknown, however, is how wings recover from disturbances and maintain their stability.

The ability of a system to recover from disturbances is often quantified by the damping capacity of that system^33^. Damping determines the energy loss that occurs during oscillations due to friction or any other resistance to movement. It is a crucial property of the wings and has both direct and indirect benefits for insect flight. On one hand, wing damping assures the production of aerodynamic forces directly by decaying unwanted wing oscillations. On the other hand, it enhances the body stability indirectly by increasing flight stability, which is essential to maintain the orientation and direction of motion in flight^34,35^. The importance of the wing damping becomes especially clear when one considers the frequency of external disturbances, which exceeds once per second in some flying insects^36^.

**Fig. 1:**
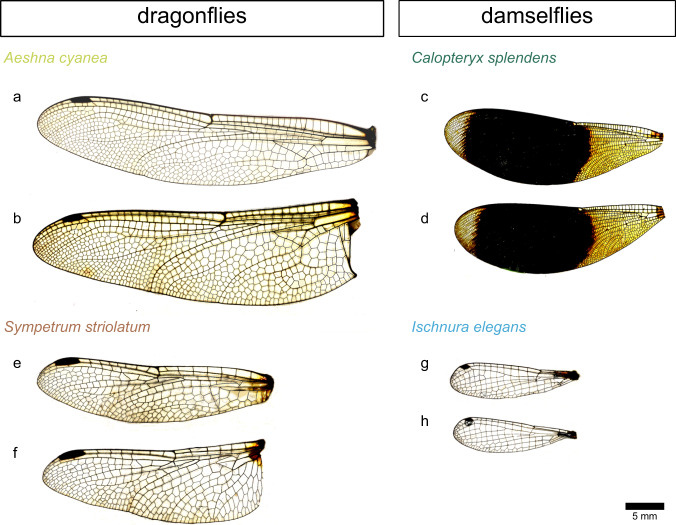
Wings of the studied species. **a**, **c**, **e**, **g** Forewings and **b**, **d**, **f**, **h** hindwings of the dragonflies *Aeshna cyanea* (**a**, **b**) and *Sympetrum striolatum* (**e**, **f**) and the damselflies *Calopteryx splendens* (**c**, **d**) and *Ischnura elegans* (**g**, **h**). Scale bar: 5 mm.

To understand the response of the wing to external disturbances, for the first time, using a viscous damping model, we characterized the damping properties of insect wings by analysing their passive return time course after a step deflection. We used dragonflies and damselflies, which are well-established model organisms for studies of insect wing biomechanics^8^, and selected the dragonflies *Aeshna cyanea* and *Sympetrum striolatum* and the damselflies *Calopteryx splendens* and *Ischnura elegans* due to their availability, wing shapes and flight styles (Fig. 1). Here we particularly aim to answer the following questions:

(i) How strongly damped are the wings?
(ii) Do different wing regions have different damping properties?
(iii) Do fore- and hindwings of dragonflies and damselflies exhibit different damping properties?
(iv) What is the role of each of the factors of the flight muscles, wing hinge, hemolymph, desiccation and rehydration in the wing damping?

This is the first comprehensive study of the wing damping. We are aware that insect wings are dynamic, living structures^37,38^ and, therefore, try to understand their properties through this lens. Knowing about the damping properties of insect wings not only helps to understand the wing response to frequent external disturbances, but is also essential to interpret dynamic shape changes of the wings during flight. Taking into account the potential role of damping in the deformation pattern of insect wings, the results are exceptionally important for future realistic modelling of insect wings by taking their damping properties into account.

## Results

**Fig. 2:**
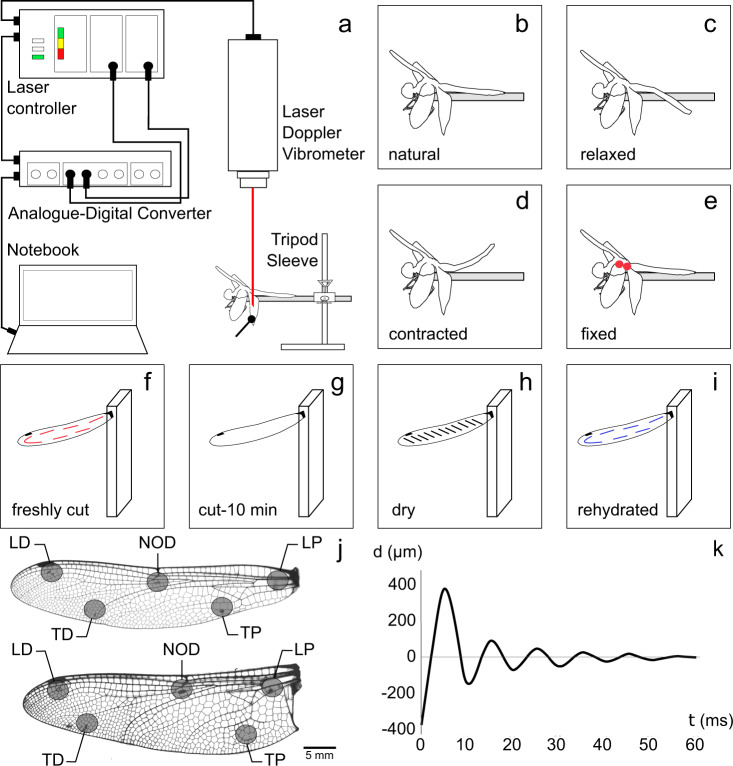
Measurement of the passive return time course of wing specimens. **a** Experimental setup. The individuals were fixed on a stiff metallic stick and a laser Doppler vibrometer was used to measure their oscillatory behaviour in different wing regions and in different treatment groups. **b**–**i** Different treatment groups included the natural (**b**), relaxed (**c**), contracted (**d**), fixed (**e**), freshly cut (**f**), cut10 min (**g**), dry (**h**) and rehydrated (**i**). **j** Measurement sites include: “leading part proximal” (LP), “nodus” (NOD), “leading part distal” (LD), “trailing part proximal” (TP), “trailing part distal” (TD). **k** Representative displacement−time curve obtained from the measurements. The results indicated that wings are underdamped.

To quantify the dynamics of the wing specimens, we first deflected them from their equilibrium state (Fig. 2a). After we released the wings, they started to oscillate about their equilibrium position. The amplitude of the oscillations decayed over time until the wings returned to rest at equilibrium (Fig. 2k). This oscillatory behaviour, which was the characteristic of all specimens, is known as underdamped oscillation.

### Comparison of damping properties of the wings at different measurement sites

**Fig. 3:**
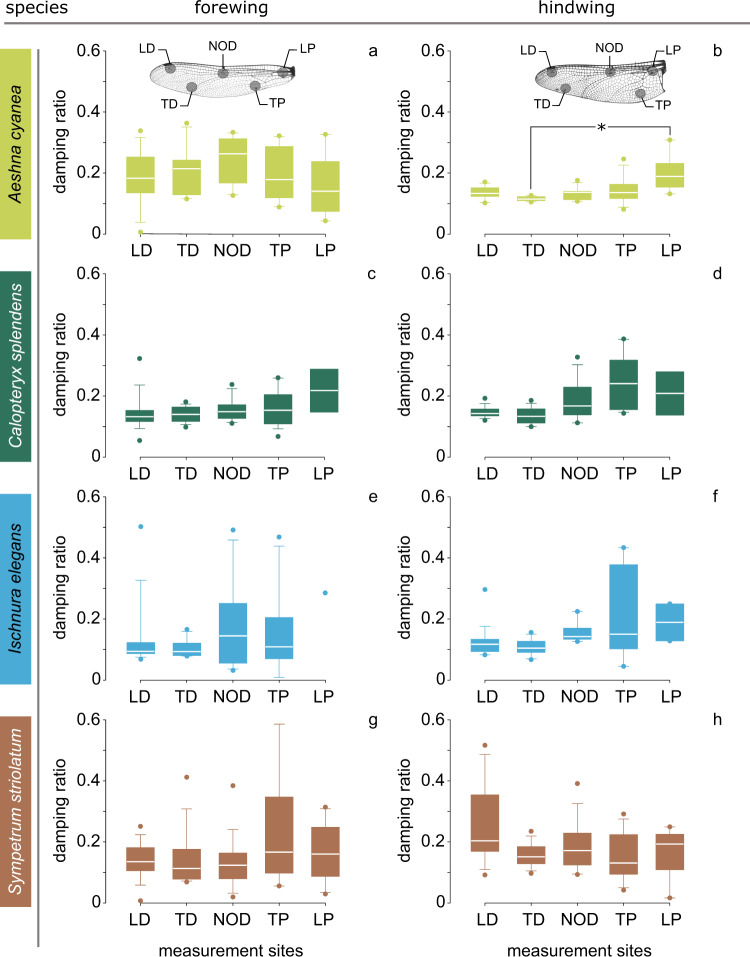
Damping ratio of freshly cut wing specimens at different measurement sites. The damping ratio is given for both forewings (**a**, **c**, **e**, **g**) and hindwings (**b**, **d**, **f**, **h**). A significant difference was found between TD and LP in the hindwing of *A. cyanea* (Friedman test, *N* = 24, *P* = 0.002), but not between the damping ratios of the other measurement sites in wings of the other examined species. LP, leading part proximal; NOD, nodus; LD, leading part distal; TP, trailing part proximal; TD, trailing part distal. In the box-and-whisker plots, the borders of the boxes indicate the 25th and 75th percentiles, the line within them marks the median, and the whiskers (error bars) define the 5th and 95th percentiles. The symbol * indicates a significant difference. The number of data points in each group is given in Table 2.

In this section, we focus on the comparison of the damping ratios of different wing regions. Fig. 3 presents the damping ratio of the freshly cut (c-f) fore- and hindwings of our model species at the different measurement sites (see Fig. 2j).

#### Aeshna cyanea

Starting with the forewing of the dragonfly *A. cyanea*, no significant difference was found between the five measurement sites (*N* = 51, *P* > 0.05, Friedman test). The damping ratio varied from 0.16 ± 0.04, in the leading part proximal (LP), to 0.25 ± 0.02, around the nodus (NOD) (Fig. 3a). In the hindwing, the maximum damping ratio, 0.20 ± 0.08, was measured in the leading part proximal (LP). This is significantly higher than the minimum damping ratio, 0.12 ± 0.02, measured in the trailing part distal (TD) (*N* = 21, *P* = 0.002, Friedman test) (Fig. 3b). No significant difference was found between the damping ratios of the other wing regions.

#### Calopteryx splendens

We found no significant difference between the damping ratios at the five measurement sites in the forewing of the damselfly *C. splendens* (*N* = 65, *P* > 0.05, ANOVA). The damping ratio varied from 0.14 ± 0.01, in the distal part proximal (TD), up to 0.22 ± 0.07, in the leading part proximal (LP) (Fig. 3c). In the hindwing, no significant difference was found between the damping ratios at different measurement sites (*N* = 64, *p* > 0.05, Friedman test). The damping ratio ranged from 0.14 ± 0.01, in the trailing part distal (TD), to 0.25 ± 0.02, in the trailing part proximal (TP) (Fig. 3d).

#### Ischnura elegans

In the forewing of the damselfly *I. elegans*, no significant difference was observed between the damping ratios of the tested wing regions (*N* = 56, *p* > 0.05, Friedman test) (Fig. 3e). The lowest damping ratio, 0.10 ± 0.01, was measured in the trailing part distal (TD) and the highest, 0.29 (only one data point available), in the leading part proximal (LP). The next highest value of the damping ratio, 0.17 ± 0.04, was found at the nodus (NOD). In hindwings, likewise, no significant difference was found between the measurement sites (*N* = 55, *p* > 0.05, Friedman test). The damping ratio varied from a minimum value of 0.11 ± 0.01, in the trailing part distal (TD), to a maximum value of 0.22 ± 0.06, in the trailing part proximal (TP) (Fig. 3f).

#### Sympetrum striolatum

We found no significant difference between damping ratios of the different measurement sites in the forewing of the dragonfly *S*. *striolatum* (*N* = 71, *p* > 0.05, Friedman test). Here the damping ratio ranged from 0.13 ± 0.02, at the nodus (NOD), to 0.23 ± 0.05, in the trailing part proximal (TP) (Fig. 3g). We did not find a significant difference between damping ratios of the different measurement sites in the hindwing (*N* = 73, *p* > 0.05, Friedman test) (Fig. 3h). The measured damping ratios varied between the highest value of 0.25 ± 0.03, in the leading part distal (LD) and the lowest value of 0.15 ± 0.02, in the trailing part proximal (TP).

For further analyses, we excluded the data points of the measurement sites that were significantly different from the others (i.e., LP in the hindwing of *A. cyanea*, Fig. 3b).

### Comparison of damping properties of the wings within and among the species

**Fig. 4:**
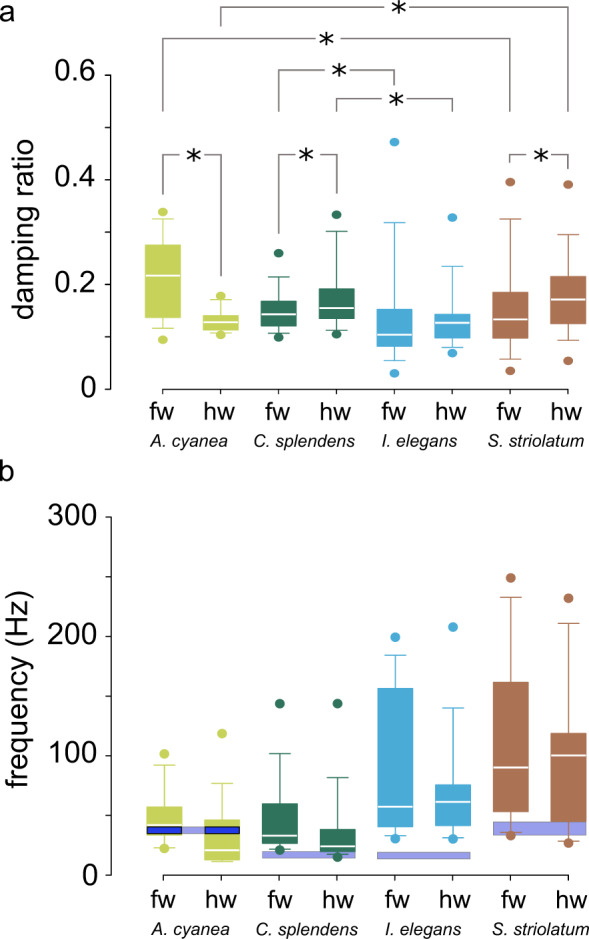
Dynamics of the freshly cut wings of the examined species. **a** Damping ratio. **b** Natural frequency. The blue bands show the range of the flapping frequencies of each species, according to the literature. fw, forewing; hw, hindwing. In the box-and-whisker plots, the borders of the boxes indicate the 25th and 75th percentiles, the line within them marks the median, and the whiskers (error bars) define the 5th and 95th percentiles. The symbol * indicates the significant difference. The number of data points in each group is given in Table 2.

The data obtained from measurements on freshly cut (c-f) wings were used to compare the oscillatory behaviour of fore- and hindwings within and among the examined species (Fig. 4).

#### Aeshna cyanea

The forewing of the dragonfly *A*. *cyanea* had a damping ratio of 0.20 ± 0.01. The damping ratio of the hindwing was found to be significantly lower than that of the forewing and equal to 0.13 ± 0.00 (*N* = 93, *p* < 0.001, Mann–Whitney test).

#### Calopteryx splendens

The fore- and hindwing of the damselfly *C*. *splendens* had a damping ratio of 0.15 ± 0.01 and 0.18 ± 0.01, respectively. A significant difference in the damping ratio was found between the wings (*N* = 129, *P* = 0.035, Mann–Whitney test).

#### Ischnura elegans

The forewing of the damselfly *I*. *elegans* had a damping ratio of 0.14 ± 0.02. The damping ratio of the hindwing, 0.14 ± 0.01, was very close to that of the forewing. We did not find a significant difference in the damping ratio between the wings (*N* = 111, *P* > 0.05, Mann–Whitney test).

#### Sympetrum striolatum

The forewing of the dragonfly *S. striolatum* had a damping ratio of 0.16 ± 0.01. The damping ratio of the hindwing was slightly higher and equal to 0.18 ± 0.01. Statistical analysis showed a significant difference in the damping ratio between the wings (*N* = 144, *P* = 0.017, Mann–Whitney test).

### Comparison of damping properties of the wings among species

Here we compare damping properties of the examined wings among the studied species. As seen in Fig. 4, in all species, except in the dragonfly *A. cyanea*, hindwings are slightly more damped than forewings. A significant difference in the damping ratio was found between the forewings and between the hindwings of the damselflies *C. splendens* and *I. elegans* (forewing: *N* = 121, *P* < 0.001, Kruskal–Wallis test; hindwing: *N* = 119, *P* < 0.001, Kruskal–Wallis test). A significant difference in the damping ratio was also found between the forewings and between the hindwings of the two studied dragonflies *A. cyanea* and *S. striolatum* (forewing: *N* = 116, *P* < 0.001, Kruskal–Wallis test; hindwing: *N* = 121, *P* < 0.001, Kruskal–Wallis test).

### Comparison of damping properties of the wings with different treatments

**Fig. 5:**
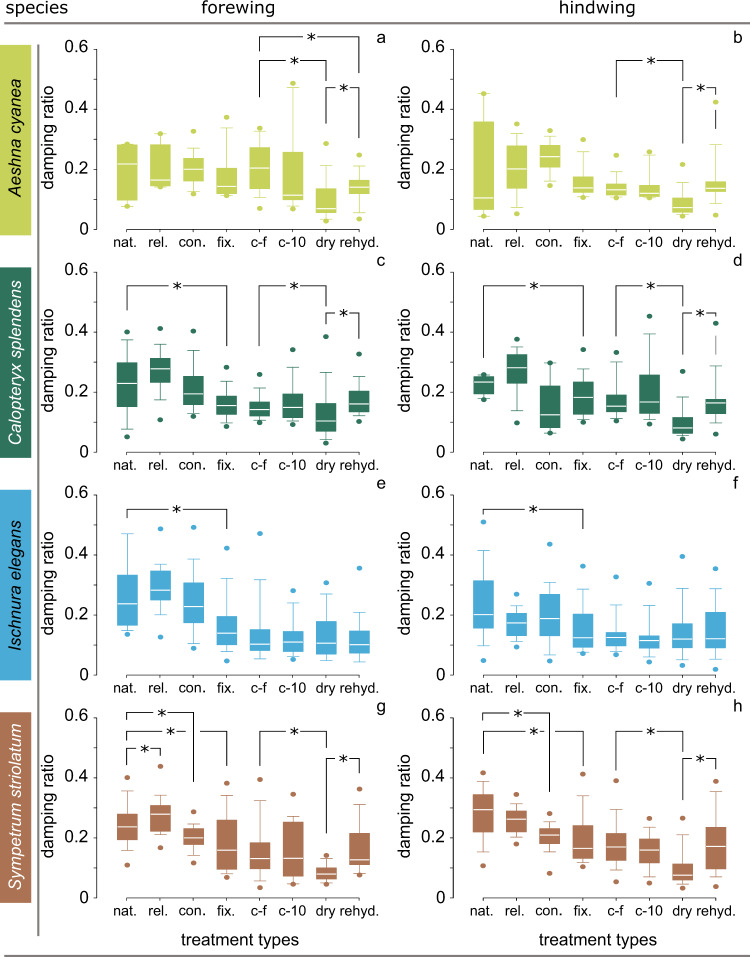
Damping ratios of differently treated specimens. The damping ratio is given for both forewings (**a**, **c**, **e**, **g**) and hindwings (**b**, **d**, **f**, **h**). Damping ratio of the wings of **a**, **b** the dragonfly *A. cyanea*; **c**, **d** the damselfly *C. splendens*; **e**, **f** the damselfly *I. elegans*; **g**, **h** the dragonfly *S. striolatum*. nat., natural wings with no treatment; rel., wings with relaxed flight muscles; con., wings with contracted flight muscles; fix., wings with fixed hinges; **c**−**f**, freshly cut wings; c-10, cut wings tested after 10 min; dry, dry wings; rehyd., rehydrated wings. In the box-and-whisker plots, the borders of the boxes indicate the 25th and 75th percentiles, the line within them marks the median, and the whiskers (error bars) define the 5th and 95th percentiles. The symbol * indicates statistically significant differences. The number of data points in each group is given in Table 2.

Fig. 5 presents the damping ratios of the differently treated wing specimens (see Fig. 2 and Methods). Here we investigate the influence of the applied treatments on the damping of fore- and hindwings of each examined species.

#### Aeshna cyanea

As seen in Fig. 5a, b, no significant difference in the damping ratio was found between the untreated (nat.) specimens and between the specimens with relaxed (rel.) and contracted (con.) muscles neither in the forewing (*N* = 33, *P* > 0.05, ANOVA) nor in the hindwing (*N* = 44, *P* > 0.05, Kruskal–Wallis test). We also found no significant difference between the untreated (nat.) specimens and those fixed at their hinge to the body (fix.) (forewing: *N* = 37, *P* > 0.05, Kruskal–Wallis test; hindwing: *N* = 57, *P* > 0.05, Kruskal–Wallis test). Similar results were obtained when comparing the damping ratios of the fixed (fix.) and freshly cut (c-f) wings (forewing: *N* = 84, *P* > 0.05, Kruskal–Wallis test; hindwing: *N* = 96, *P* > 0.05, Kruskal–Wallis test). Comparison of the freshly cut (c-f) wings and wings left for 10 min after separation from body (c-10) also resulted in no significant difference (forewing: *N* = 65, *P* > 0.05, Kruskal–Wallis test; hindwing: *N* = 56, *P* > 0.05, Kruskal–Wallis test). The desiccated specimens (dry), however, had significantly lower damping ratios than the freshly cut (c-f) specimens (forewing: *N* = 117, *P* < 0.001, Kruskal–Wallis test; hindwing: *N* = 110, *P* < 0.001, Kruskal–Wallis test). The rehydration significantly increased the damping ratio of the dry wings (forewing: *N* = 80, *P* = 0.017, Kruskal–Wallis test; hindwing: *N* = 74, *P* < 0.001, Kruskal–Wallis test). Comparison of the rehydrated (rehyd.) wings with the freshly cut (c-f) wings resulted in a significant difference in the forewings (*N* = 65, *P* = 0.011, Kruskal–Wallis test), but not in the hindwings (*N* = 60, *P* > 0.05, Kruskal–Wallis test).

#### Calopteryx splendens

Fig. 5c, d presents the damping ratio of the wings of *C. splendens*. Comparison of the damping ratios of the untreated (nat.) specimens with those in the relaxed (rel.) and contracted (cont.) sample treatment groups showed no significant difference (forewing: *N* = 79, *P* > 0.05, Kruskal–Wallis test; hindwing: *N* = 42, *P* > 0.05, ANOVA). However, the damping ratio of the untreated (nat.) wings was significantly higher than that of the wings with fixed hinges (fix.) (forewing: *N* = 92, *P* = 0.002, Kruskal–Wallis test; hindwing: *N* = 69, *P* = 0.029, Kruskal–Wallis test). No significant difference was found in the damping ratio between the fixed (fix.) and freshly cut (c-f) wings (forewing: *N* = 115, *P* > 0.05, Kruskal–Wallis test; hindwing: *N* = 121, *P* > 0.05, Kruskal–Wallis test). Comparison of the damping ratios of the freshly cut (c-f) wings and those tested 10 min after cutting off (c-10) also showed no significant difference (forewing: *N* = 84, *P* > 0.05, Kruskal–Wallis test; hindwing: *N* = 83, *P* > 0.05, Kruskal–Wallis test). Dry wings (dry) had a significantly lower damping ratio in comparison with the freshly cut (c-f) wings (forewing: *N* = 135, *P* < 0.001, Kruskal–Wallis test; hindwing: *N* = 132, *P* < 0.001, Kruskal–Wallis test). Comparing the damping ratio of the dry wings (dry) with the rehydrated (rehyd.) ones also showed a significant difference (forewing: *N* = 88, *P* = 0.002, ANOVA; hindwing: *N* = 84, *P* < 0.001, Kruskal–Wallis test). Comparing the rehydrated (rehyd.) wings with the freshly cut (c-f) wings showed no significant differences in the forewings (*N* = 83, *P* > 0.05, Kruskal–Wallis test) and in the hindwings (*N* = 80, *P* > 0.05, Kruskal–Wallis test).

#### Ischnura elegans

After comparing the results obtained from the differently treated wings of the damselfly *I. elegans*, a significant difference was found only between the damping ratio of the untreated (nat.) specimens and those that were fixed at their hinge (fix.) (forewing: *N* = 71, *P* < 0.001, Kruskal–Wallis test; hindwing: *N* = 79, *P* < 0.001, Kruskal–Wallis test) (Fig. 5e, f). Statistical analysis showed no significant difference between the damping ratios of the untreated (nat.) specimens and those with relaxed (rel.) and contracted (con.) muscles (forewing: *N* = 86, *P* > 0.05, Kruskal–Wallis test; hindwing: *N* = 109, *P* > 0.05, Kruskal–Wallis test). No significant difference was found in the damping ratio between the fixed (fix.) wings and the freshly cut (c-f) wings (forewing: *N* = 101, *P* > 0.05, Kruskal–Wallis test; hindwing: *N* = 97, *P* > 0.05, Kruskal–Wallis test). No significant difference was also found in the damping ratio between the freshly cut (c-f) wings and those left for 10 min after separation from body (c-10) (forewing: *N* = 92, *P* > 0.05, Kruskal–Wallis test; hindwing: *N* = 90, *P* > 0.05, Kruskal–Wallis test). Surprisingly, the damping ratio of the wings did not change significantly after desiccation (forewing: *N* = 124, *P* > 0.05, Kruskal–Wallis test; hindwing *N* = 134, *P* > 0.05, Kruskal–Wallis test). Also, the damping properties of the rehydrated specimens (rehyd.) did not significantly change compared to the dry (dry) specimens (forewing: *N* = 101, *P* > 0.05, Kruskal–Wallis test; hindwing: *N* = 120, *P* > 0.05, Kruskal–Wallis test). Comparing the rehydrated (rehyd.) wings with the freshly cut (c-f) wings showed no significant difference in the forewings (*N* = 89, *P* > 0.05, Kruskal–Wallis test) and no difference in the hindwings (*N* = 96, *P* > 0.05, Kruskal–Wallis test).

#### Sympetrum striolatum

Fig. 5g, h presents the damping ratio of the fore- and hindwings of the dragonfly *S. striolatum* in each treatment group. Comparison of the damping ratios of the untreated (nat.) specimens and those in the contracted (cont.) and relaxed (rel.) treatment groups showed a difference in the forewing (*N* = 150, *P* < 0.001, Kruskal–Wallis test). In the hindwing, the damping ratio of untreated wings (0.28 ± 0.01) was significantly higher than that of the wings with contracted muscles (0.20 ± 0.01) (*N* = 83, *P* < 0.001, Kruskal–Wallis test). The damping ratio of the untreated fore- and hindwings (nat.) significantly decreased after fixing their hinge with the body (forewing: *N* = 157, *P* < 0.001, Kruskal–Wallis test; hindwing: *N* = 150, *P* < 0.001, Kruskal–Wallis test). No significant difference was found in the damping ratio between the wings with fixed hinges (fix.) and the freshly cut (c-f) wings (forewing: *N* = 129, *P* > 0.05, Kruskal–Wallis test; hindwing: *N* = 170, *P* > 0.05, Kruskal–Wallis test). No significant difference was also found between the damping ratios of the freshly cut (c-f) wings and those tested after 10 min (c-10) (forewing: *N* = 90, *P* > 0.05, Kruskal–Wallis test; hindwing: *N* = 177, *P* > 0.05, Kruskal–Wallis test). While desiccation significantly decreased the damping ratio of the freshly cut (c-f) wings (forewing: *N* = 124, *P* < 0.001, Kruskal–Wallis test; hindwing: *N* = 143, *P* < 0.001, Kruskal–Wallis test), rehydration significantly increased the damping ratio of the desiccated wings (dry) (forewing: *N* = 93, *P* < 0.001, Kruskal–Wallis test; hindwing: *N* = 90, *P* < 0.001, Kruskal–Wallis test). Comparing the rehydrated (rehyd.) wings with the freshly cut (c-f) wings showed no significant difference between the forewings (*N* = 111, *P* > 0.05, Kruskal–Wallis test) and between the hindwings (*N* = 93, *P* > 0.05, Kruskal–Wallis test).

Table 1 summarizes the effects of the different sample treatments on the damping ratio of the fore- and hindwings of the examined species.

**Table 1:** Comparison between damping ratios of wings in the different treatment groups.

Comparison		nat. vs. rel.	nat vs. con.	natural vs. fix.	fixed vs. c(f)	c(f) vs. c(10)	c(f) vs. dry	dry vs. rehyd.	c(f) vs. rehyd.
		*natural* vs. *relaxed*	*natural* vs. *contracted*	*natural* vs. *fixed*	*fixed* vs. *freshly cut*	*freshly cut* vs. *10* *min cut*	*freshly cut* vs. *dry*	*dry* vs. *rehydrated*	*freshly cut* vs. *rehydrated*
Influencial factor		muscle	muscle	wing hinge	hemolymph pressure	hemolymph presence	desiccation	solution influence	solution influence
species	wing								
*Aeshna cyanea*	fw	×	×	×	×	×	✓	✓	✓
	hw	×	×	×	×	×	✓	✓	×
*Calopteryx splendens*	fw	×	×	✓	×	×	✓	✓	×
	hw	×	×	✓	×	×	✓	✓	×
*Ischnura elegans*	fw	×	×	✓	×	×	×	×	×
	hw	×	×	✓	×	×	×	×	×
*Sympetrum striolatum*	fw	✓	✓	✓	×	×	✓	✓	×
	hw	×	✓	✓	×	×	✓	✓	×

The symbols ✓ and × indicate the presence and absence of a significant difference between the two treatment groups, respectively. *fw* forewing, *hw* hindwing.

### Natural frequency and flapping frequency of the wings

Fig. 4b presents the natural frequencies of the freshly cut (c-f) fore- and hindwings of the examined species. The blue bands show the range of the flapping frequencies of each species obtained from the literature^39–42^.

#### Aeshna cyanea

The forewings of *A. cyanea* had a natural frequency of 48.0 ± 3.4 Hz. The hindwings showed a lower natural frequency of 32.1 ± 4.3 Hz in comparison to the forewings. The flapping frequency of the wings varies between 36.0 Hz and 40.0 Hz^39,40^.

#### Calopteryx splendens

The natural frequency of the forewings of *C. splendens* was 49.6 ± 4.6 Hz. The natural frequency of the hindwing was lower and equal to 36.8 ± 4.3 Hz. According to Rüppel^39^, Grabow & Rüppel^40^, and Wakeling & Ellington^41^, the wings of this species have a flapping frequency ranging between 16.0 and 20.0 Hz.

#### Ischnura elegans

The forewings of *I. elegans* had a natural frequency of 100.6 ± 12.3 Hz. The hindwings had a natural frequency of 78.5 ± 8.8 Hz. The flapping frequency of the wings ranges between 15.3 and 20.7 Hz^42^ and is markedly lower in comparison with the measured natural frequencies.

#### Sympetrum striolatum

The fore- and hindwings of *S. striolatum* had a natural frequency of 116.9 ± 9.4 Hz and 96.6 ± 7.2 Hz, respectively. Although we did not find any data in the literature on the flapping frequencies of *S. striolatum*, those of the closely related species *S. sangineum*, *S. danae* and *S. vulgatum*, range between 32.3 and 43.5 Hz^39,41^.

The results of all the performed statistical analyses in this study are summarized in Table 2.

**Table 2:** Summary of the results of the dynamics of wings of the examined species.

	*A. cyanea* fw	*A. cyanea* hw	*C. splendens* fw	*C. splendens* hw	*I. elegans* fw	*I. elegans* hw	*S. striolatum* fw	*S. striolatum* hw
*Damping ratio at different measurement sites (treatment c-f)*								
LD leading distal	*N* = 12	*N* = 12	*N* = 16	*N* = 16	*N* = 14	*N* = 17	*N* = 16	*N* = 16
	**0.188** **±** **0.025**	**0.135** **±** **0.0059**	**0.143** **±** **0.0142**	**0.147** **±** **0.0045**	**0.129** **±** **0.0293**	**0.214** **±** **0.0119**	**0.141** **±** **0.0143**	**0.251** **±** **0.0318**
	*0.183*	*0.133*	*0.132*	*0.143*	*0.0934*	*0.117*	*0.134*	*0.203*
TD trailing distal	*N* = 11	*N* = 12	*N* = 16	*N* = 16	*N* = 14	*N* = 20	*N* = 14	*N* = 16
	**0.206** **±** **0.0242**	**0.115** **±** **0.002**	**0.140** **±** **0.0061**	**0.135** **±** **0.0067**	**0.104** **±** **0.0076**	**0.109** **±** **0.0061**	**0.142** **±** **0.0239**	**0.157** **±** **0.0097**
	*0.216*	*0.114*	*0.140*	*0.134*	*0.0938*	*0.105*	*0.114*	*0.151*
NOD nodus	*N* = 12	*N* = 12	*N* = 14	*N* = 16	*N* = 14	*N* = 9	*N* = 16	*N* = 16
	**0.246** **±** **0.0212**	**0.133** **±** **0.0058**	**0.155** **±** **0.0098**	**0.187** **±** **0.0156**	**0.172** **±** **0.0378**	**0.155** **±** **0.0105**	**0.130** **±** **0.0205**	**0.185** **±** **0.0198**
	*0.264*	*0.137*	*0.148*	*0.168*	*0.144*	*0.143*	*0.124*	*0.171*
TP trailing proximal	*N* = 10	*N* = 12	*N* = 17	*N* = 14	*N* = 13	*N* = 7	*N* = 14	*N* = 16
	**0.197** **±** **0.0267**	**0.142** **±** **0.0121**	**0.161** **±** **0.0136**	**0.248** **±** **0.0237**	**0.154** **±** **0.0384**	**0.220** **±** **0.0576**	**0.233** **±** **0.0500**	**0.148** **±** **0.0197**
	*0.180*	*0.137*	*0.153*	*0.241*	*0.109*	*0.150*	*0.167*	*0.131*
LP leading proximal	*N* = 6	*N* = 9	*N* = 2	*N* = 2	*N* = 1	*N* = 2	*N* = 11	*N* = 9
	**0.158** **±** **0.0411**	**0.199** **±** **0.0184**	**0.218** **±** **0.0705**	**0.209** **±** **0.0711**	**0.285**	**0.189** **±** **0.0605**	**0.170** **±** **0.0289**	**0.157** **±** **0.0275**
	*0.141*	*0.189*	*0.218*	*0.209*	*0.285*	*0.189*	*0.160*	*0.193*
*Damping ratio of the wings*								
Merged wings regions	*N* = 45^a^	*N* = 48^a^	*N* = 65	*N* = 64	*N* = 56	*N* = 55	*N* = 71	*N* = 73
	**0.210** **±** **0.0122**	**0.131** **±** **0.00385**	**0.152** **±** **0.00597**	**0.178** **±** **0.00868**	**0.142** **±** **0.0152**	**0.138** **±** **0.00975**	**0.161** **±** **0.0135**	**0.183** **±** **0.0107**
	*0.216*	*0.126*	*0.142*	*0.154*	*0.103*	*0.126*	*0.132*	*0.170*
*Damping ratio of the wings in different treatment groups*								
Natural	*N* = 4	*N* = 9^a^	*N* = 42	*N* = 12	*N* = 26	*N* = 37	*N* = 99	*N* = 53
	**0.199** **±** **0.0495**	**0.189** **±** **0.0529**	**0.226** **±** **0.0162**	**0.224** **±** **0.00844**	**0.275** **±** **0.0271**	**0.237** **±** **0.0201**	**0.242** **±** **0.00784**	**0.280** **±** **0.0124**
	*0.217*	*0.105*	*0.229*	*0.234*	*0.238*	*0.202*	*0.237*	*0.294*
Relaxed	*N* = 4	*N* = 20^a^	*N* = 23	*N* = 25	*N* = 32	*N* = 46	*N* = 19	*N* = 18
	**0.197** **±** **0.0410**	**0.208** **±** **0.0197**	**0.272** **±** **0.0150**	**0.270** **±** **0.0150**	**0.291** **±** **0.0141**	**0.173** **±** **0.00802**	**0.279** **±** **0.00784**	**0.260** **±** **0.0102**
	*0.164*	*0.201*	*0.278*	*0.282*	*0.283*	*0.174*	*0.279*	*0.262*
Contracted	*N* = 25	*N* = 15^a^	*N* = 14	*N* = 5	*N* = 28	*N* = 26	*N* = 32	*N* = 28
	**0.200** **±** **0.0107**	**0.239** **±** **0.0128**	**0.208** **±** **0.0196**	**0.146** **±** **0.0402**	**0.241** **±** **0.0201**	**0.200** **±** **0.0189**	**0.202** **±** **0.00743**	**0.204** **±** **0.00852**
	*0.200*	*0.242*	*0.194*	*0.125*	*0.228*	*0.189*	*0.201*	*0.209*
Fixed	*N* = 33	*N* = 48^a^	*N* = 50	*N* = 57	*N* = 45	*N* = 42	*N* = 58	*N* = 97
	**0.177** **±** **0.0144**	**0.160** **±** **0.00835**	**0.165** **±** **0.00925**	**0.189** **±** **0.00875**	**0.167** **±** **0.0155**	**0.159** **±** **0.0157**	**0.186** **±** **0.0136**	**0.201** **±** **0.0104**
	*0.143*	*0.140*	*0.156*	*0.183*	*0.140*	*0.125*	*0.159*	*0.165*
Freshly cut	*N* = 51	*N* = 48^a^	*N* = 65	*N* = 64	*N* = 56	*N* = 55	*N* = 71	*N* = 73
	**0.204** **±** **0.0119**	**0.131** **±** **0.00385**	**0.152** **±** **0.00597**	**0.178** **±** **0.00868**	**0.142** **±** **0.0152**	**0.138** **±** **0.00975**	**0.161** **±** **0.0135**	**0.183** **±** **0.0107**
	*0.205*	*0.126*	*0.142*	*0.154*	*0.103*	*0.126*	*0.132*	*0.170*
10 min cut	*N* = 14	*N* = 8^a^	*N* = 19	*N* = 19	*N* = 36	*N* = 35	*N* = 19	*N* = 104
	**0.188** **±** **0.0376**	**0.119** **±** **0.00459**	**0.167** **±** **0.0150**	**0.201** **±** **0.0225**	**0.128** **±** **0.0115**	**0.132** **±** **0.0146**	**0.157** **±** **0.0202**	**0.158** **±** **0.00613**
	*0.114*	*0.105*	*0.150*	*0.168*	*0.110*	*0.115*	*0.132*	*0.160*
Dry	*N* = 66	*N* = 62^a^	*N* = 70	*N* = 68	*N* = 68	*N* = 79	*N* = 53	*N* = 70
	**0.106** **±** **0.0101**	**0.0865** **±** **0.00838**	**0.133** **±** **0.0115**	**0.102** **±** **0.00797**	**0.132** **±** **0.0100**	**0.147** **±** **0.0109**	**0.0852** **±** **0.00427**	**0.102** **±** **0.00983**
	*0.0687*	0.0675	*0.104*	*0.0814*	*0.107*	*0.120*	*0.0802*	*0.0760*
Rehydrated	*N* = 14	*N* = 12^a^	*N* = 18	*N* = 16	*N* = 33	*N* = 41	*N* = 40	*N* = 20
	**0.138** **±** **0.0130**	**0.140** **±** **0.00545**	**0.175** **±** **0.0129**	**0.170** **±** **0.0197**	**0.123** **±** **0.0153**	**0.149** **±** **0.0140**	**0.168** **±** **0.0140**	**0.180** **±** **0.0217**
	*0.140*	*0.137*	*0.161*	*0.165*	*0.102*	*0.122*	*0.127*	*0.172*
*Natural frequency (Hz)*								
Freshly cut wings	*N* = 51	*N* = 57	*N* = 65	*N* = 64	*N* = 56	*N* = 55	*N* = 71	*N* = 73
	**47.971** **±** **3.381**	**32.103** **±** **4.264**	**49.593** **±** **4.559**	**36.751** **±** **4.247**	**100.572** **±** **12.282**	**78.486** **±** **8.835**	**116.846** **±** **9.373**	**96.567** **±** **7.179**
	*40.620*	*19.668*	*31.528*	*22.870*	*65.986*	*60.392*	*89.223*	*99.263*

Number of data points (*N*); mean ± standard error are shown in bold and median values in italics.

*fw* forewing, *hw* hindwing.

^a^Values without LP.

## Discussion

Odonata wings, similar to wings of many other flying insects, consist of supporting and deformable regions, which exhibit different mechanical behaviours^23^. Taking into account that there is no clear border between the wing regions, to understand whether the damping properties vary between the regions, we subdivided the wings of our model species into five measurement sites (Fig. 2j). Considering the distribution of the measurement sites, we expected them to capture any potential difference in the damping properties between the leading edge spar and trailing region as well as between the proximal and distal parts of the wings. Surprisingly, except in one case (i.e., LP in the hindwings of the dragonfly *A. cyanea*, Fig. 3b), the damping ratio of the wings was the same at all measurement sites. This is an interesting finding which may imply that, regardless of their different structural (and perhaps material) characteristics, the different wing regions are almost equally damped. Thus, according to their damping properties, the wings are likely to be homogeneous.

The mean damping ratio of the examined wings in this study ranged from 0.13 to 0.20 for the freshly cut (c-f) specimens (this was slightly higher for the untreated specimens, ranging from 0.18 to 0.28). The wing damping ratio is smaller than those of owl feathers^43^ (i.e., 0.20–0.32), human body^44^ (i.e., 0.3–0.5) and cartilage^44^ (i.e., 0.30). In contrast, this is noticeably higher than those of many civil engineering structures^45^ (i.e., 0.01–0.02), concrete dams/bridges^46,47^ (i.e., 0.02–0.05) and even that of pigeon feathers^43^ (i.e., 0.05–0.08). Interestingly, the wing damping ratio is very close to that measured for olive tree trunks^48^ (i.e., 0.17–0.2).

The damping ratios obtained in this study are a few times higher than the only existing data in the literature on the damping ratio of insect wings measured by Chen et al.^49^ for the forewings of the dragonfly species *Orthetrum pruinosum* and *O. sabina*, which is 0.05. This difference can be attributed to the fact that Chen et al.^49^ examined desiccated wings. However, as seen in our experiments, desiccation can significantly reduce the wing damping ratio. This explains why the previously reported damping ratio is close to the lowest mean damping ratio obtained in our study (i.e., 0.09 for the dry forewings of *S. striolatum*).

Why do wings need to be damped? The answer is that, similar to any other natural or engineering vibrating system, damping plays a key role in the dynamics of insect wings by providing them with stability. The role of damping in the dynamic response of a system can be exceptionally significant when contact events are involved^50^, which is usually the case for insect wings. According to the previous reports, wings of flying insects frequently experience contact events (e.g., collisions with vegetation), under which they often undergo large deformations^13,24,26,36^. To minimize their impact on the aerodynamic performance of insects, wings should rapidly return to their original posture. This can be achieved if the wings are damped enough.

What are the origins of the wing damping and why does the damping ratio vary among the examined wings? Previous studies have reported conflicting results regarding the role of aerodynamic damping in the wing dynamics. Although Combes and Daniel^51^ suggested the presence of only a minimal external damping by the air resistance, Norris et al.^52^ showed that aerodynamics may influence the wing effective damping. Despite this contradictory finding, the material and structure are certainly two key sources of damping in insect wings^51,52^. The difference in the damping ratios of the wings can, therefore, be attributed to their different material properties and structural design. Although all insect wings are made of cuticle, the properties of the wing cuticle can largely vary from one to another species^53,54^. In addition to wing material properties, the wing shape and structure also show noticeable variations among different species^13^. The difference in the structural design is also obvious, especially when comparing fore- and hindwings of the examined dragonflies, and can explain their significantly different damping properties. The same argument can be used to justify the absence of a significant difference between the damping ratios of the fore and hindwings of *I. elegans* and only a weak difference in *C. splendens* that have nearly identical fore- and hindwings.

Considering that the wing damping is essential for flight, why are not the wings more strongly damped than they are? The answer may be found in the conflict between stiffness and damping. Although researches have used a variety of strategies to combine stiffness and damping in engineered systems, these two properties are often mutually exclusive^55^. Insect wings should be stiff enough to withstand aerodynamic forces. Any increase of the damping ratio of the wings comes with a sacrifice of the stiffness. Hence, a balance between the two properties is needed to achieve a fully functional wing^11^. The current damping ratio of the wings is thought to facilitate this balance. A quantitative investigation of the trade-offs between stiffness and damping of the wings of different insect species is, in our opinion, a promising direction for future research.

Energetic efficiency is another factor that can limit the damping capacity of the flight system in insects. As shown for the hawkmoth *Manduca sexta*, structural damping of the thorax can be a substantial source for energy dissipation^56^. The energy loss, due to damping, can consequently increase the power required for flapping-wing motion. A similar effect can be assumed for the wing damping. This further highlights the importance of understanding the damping properties of insect wings for insect flight energetics.

Odonata, to a great extent, owe their impressive flight to their ability to move the wings independently from each other^57^. This ability is achieved by their so-called direct flight muscles that are directly connected to wing base sclerites^58,59^. To investigate any potential contribution of the flight muscles to the damping properties of the wings, we compared the damping ratio of our untreated specimens with those that had relaxed or contracted muscles (nat. vs. rel./con., Fig. 5). The absence of a significant difference in the damping ratio between the mentioned treatment groups (except for the forewing of *S. striolatum*) suggests that, surprisingly, muscles do not play a dominant role in the wing damping.

What about the role of the wing hinge? In Odonata, the wings are hinged to the body via the anterior humeral and posterior axillary plates^60,61^. To assess the influence of the wing-body hinge on the wing damping, we compared the damping ratios of the untreated specimens and those with the fixed hinges (nat. vs. fix., Fig. 5). In all species, except in *A. cyanea*, the wing hinge was found to significantly contribute to the wing damping. This is likely caused by the elastic resilin-rich cuticle in the wing hinge, similar to that described by Weis-Fogh^62^ in locusts. This observation confirms the importance of the wing hinge in the wing function that has already been pointed out in some former studies^60,61,63^.

The absence of the influence of the wing hinge on the wing damping in *A. cyanea* may suggest a difference in the wing hinge architecture/properties between flier and percher Odonata; *A. cyanea* is a flier dragonfly and the other three examined species are perchers^64^. Fliers are known for their fast continuous flights, while perchers make short regular flights around perches^65^. The different flight styles of the two groups may be linked to the role of the wing hinge in their different damping properties. The mechanisms behind this difference, however, remain to be explored.

Most veins in dragonfly and damselfly wings are filled with hemolymph, which circulates through the vein network^66^. It supplies the wing cuticle with water, nutrients, and other substances and further removes wastes^67^. The influence of hemolymph on the damping properties of the wings was studied here by comparing the results obtained from the wing specimens that were fixed to the body and those tested immediately and 10 min after removal from the body (fix. vs. c-f/c-10, Fig. 5). While the former comparison (i.e., fix. vs. c-f) was expected to show the influence of hemolymph pressure on the wing damping, the latter (i.e., fix. vs. c-10) was used to explicitly address the effect of the hemolymph presence. The absence of a significant change in the damping ratio of the wings after being cut from the body (tested either immediately or after a 10 min delay) indicated no direct influence of hemolymph on the damping of the dragonfly and damselfly wings.

The results of our experiments are in contradiction with those obtained from the theoretical solution of Wang and Zhong^68^. In their study, using a simplified model of an elastic tube, Wang and Zhong^68^ showed that hemolymph flow can increase the damping of their model. Using the argument that wing veins in dragonflies are also tubular and convey a flowing fluid, they concluded that the same effect can be expected in the whole wing. However, in insect wings and particularly in dragonfly wings, hemolymph circulates in a complex network of interconnected veins^66^. This means that, while hemolymph in a vein flows towards the wingtip, in an adjacent vein it may move in an opposite direction. Therefore, the effect of the hemolymph flow in one vein could be counterbalanced by that in another vein. It is likely that the simplified model of Wang and Zhong^68^ is not able to take this complexity into account.

The results of our experiments, when extended to other species, need to be interpreted with caution as they may not be valid for all insects. The wings of some small insects, such as mosquitos may be highly affected by the loss of hemolymph. Although here we did not find a direct effect of hemolymph on the wing damping, we cannot fully exclude the presence of such effects. It may be the case that hemolymph has an effect in flapping and dynamic flight, which could not be captured here. In 1972, Norberg^69^ showed that the pterostigma, which is actually a box-like hemolymph sinus close to the wing tip^66,69,70^, enhances the stability of flapping wings by reducing feathering vibrations. However, we expect this to be more an indirect effect, especially considering that Norberg^69^ related his findings to the greater mass of the pterostigma, in comparison to other wing regions. It may also be the case that the 10-min time period in our experiments was not enough to empty the wing of hemolymph, especially considering their complex vein networks. Cutting off the wings also results in the removal of the pump system that pulls hemolymph back into the body. More realistic computational simulations may help to shed more light on the role of hemolymph on the response of insect wings to applied loads. For precise simulations of the mechanical behaviour of insect wings, further studies on the wing circulation and vein shape are needed^37,67^.

Previous studies have investigated the effect of desiccation on the stiffness of the cuticle of different insect body parts^71,72^. To find out how desiccation influences the damping properties of the wing cuticle, we compared the results of our experiments on freshly cut and dry specimens (c-f vs. dry, Fig. 5). The observed decrease in the damping ratio of the wings of the examined species, except *I. elegans*, can be explained by the increase of the cuticle stiffness after desiccation^73,74^. When desiccation takes place, the endocuticle, which is the most hydrated part of cuticle, is affected more than any other cuticle layers. By losing its water, the endocuticle becomes almost as stiff as the typically dehydrated exocuticle^71,72^. The stiffening of the endocuticle consequently leads to an increase in the stiffness of the whole wing system, which can result in the observed decrease in the damping ratio of the wings.

The results of our desiccation experiments suggest the indirect role of hemolymph in the wing damping. The fact is that the cuticle of insect wings is a living material. To keep it alive, it should be hydrated continuously. As mentioned earlier, this task is accomplished by hemolymph, which flows within the wing veins. Although our results did not show the direct influence of hemolymph on the wing damping, this does not mean that hemolymph does not influence the damping properties of the wings. In fact, based on our results, hemolymph influences the wing damping indirectly by continuously hydrating the wing system, which is essential for maintaining wing properties, in particular the wing damping ratio.

Our results can also be used to determine the potential influence of mechanical damages on the wing damping. As indicated by previous studies, wing damage, particularly in form of area loss, often occurs at the wingtip and trailing edge^25,28,30,75^. Such damages, which are mainly caused by mechanical collisions, significantly increase over time and as an insect ages^28^. The wing damage can disturb the hemolymph circulation in the wing and, consequently, result in desiccation of the wing material. Considering the influence of desiccation on the wing damping observed in this study, we expect the wing damping to decrease over the lifespan of a flying insect.

Why desiccation did not change the damping ratio of the wings in *I. elegans* remains unclear. A simple explanation could be the fast desiccation due to the small size of the wings. Considering that desiccation starts quickly after cutting off the wings, the preparation time for testing the freshly cut (c-f) specimens could have been long enough to cause the desiccation of the relatively small wings of *I. elegans*. Although this may explain why no significant difference was observed between the damping ratio of the freshly cut and the dry specimens, it does not explain why after rehydration the damping ratio still remained unchanged (Fig. 5e, **f**).

Previous studies have frequently used rehydrated specimens to measure the material properties, in particular the elastic modulus, of insect cuticle^71,76–79^. These studies were based on the assumption that alteration of water content can reproducibly change cuticle properties. Our results showed that the same is valid for the effect of the rehydration on the dynamics of insect cuticle; except for *I. elegans*, the damping ratio of dry wing specimens significantly increased after rehydration (dry vs. rehyd. Fig. 5).

Can rehydration fully restore the material properties of the cuticle after desiccation? To answer this question, we compared the damping ratios of the rehydrated wings versus those of the freshly cut wings (rehyd. vs. c-f, Fig. 5). We found that, except for the forewing of *A. cyanea*, no significant difference existed between the two sets of data. Hence, according to our results, we suggest that rehydrated specimens can be reliably used to measure the damping properties of insect cuticle.

One should still take into account that our specimens were dehydrated for only about 24 h. Hence, it remains unclear how rehydration can restore the damping properties of museum specimens or those kept in dry conditions for longer periods of time. Future studies should investigate the effect of longer desiccation times on the damping properties of rehydrated wings.

Previous studies have reported conflicting data regarding the relationship between the flapping frequency and the natural frequency of insect wings. Although some studies have shown that insects have flapping frequencies below the natural frequency of their wings^49,80–82^, some others argued that, to save energy, insects flap at frequencies near or equal to their wing natural frequencies^26,83^. Interestingly, here we found both of these relationships between the flapping and natural frequencies in the wings of our examined species (Fig. 4b). While the flier *A. cyanea* exhibited flapping frequencies almost equal to the natural frequency of its wings, the three other percher species flap at frequencies below their wing natural frequencies. Although it is reasonable to hypothesize that *A. cyanea*, due to the considerable amount of time it spends in flight, has developed strategies for energy saving, whether this difference is really influenced by the specific flight behaviour of the studied species requires further investigations.

As mentioned earlier, in contrast to birds and bats, insects have no or only a minor direct control on their wing shape changes in flight. Hence, shape changes of insect wings, which are often very remarkable, are mainly influenced by the wing architecture and material properties. For many years, researchers have been aiming to explore the link between passive shape changes of insect wings and their aerodynamic performance^9,11,13,16^. An important step in this direction is to understand the structural and biomechanical background of the wing deformations. Taking into account the importance of the damping on the dynamic response of the wings, our results can be used to better interpret the mechanical behaviour of wings at various loading regimes. This will be particularly possible with the development of realistic models of insect wings by taking wing damping into account. We expect our study to facilitate future studies on insect wing biomechanics.

Four directions for future research seem particularly worth following. First, a quantitative comparison of wing dynamics between insects with different flight styles would be an interesting area for further investigations. Although our results suggest the presence of a potential relationship between wing dynamics and flight style of the examined dragonfly species, we cannot extend this relationship to other insects. Second, we require information on the vibration mode shapes of insect wings. This is because any mode shape may have a unique damping ratio and also a unique natural frequency. Although here we examined the damping ratio of the wings in their dominant vibration mode, wings may have multiple damping ratios that correspond to multiple modes of vibration. Third, future studies can investigate the efficiency of other damping models (e.g., coulomb, hysteretic, etc.) than the viscous damping model used here for describing the wing damping. Although the viscous damping model is the most common approach for modelling the dynamic response of biological systems^74,84,85^, it might not always be the best model for characterizing their dynamic behaviour. This has previously shown to be the case for the thorax of the hawkmoth *Manduca sexta*^56^ or the hindleg of the cockroach *Blaberus discoidalis*^86^. Finally, there is a need for understanding the extent of the nonlinearity of wing vibrations. It is not fully clear whether insect wings behave linearly, as assumed here. Considering that vibratory systems may exhibit nonlinear behaviour with increasing amplitude of oscillations, a knowledge of linear/nonlinear vibrations is desirable for detailed characterization of the wing damping. Future research is expected to shed more light on the dynamics of complicated insect wings.

## Conclusions

Based on the results obtained from non-contact measurements of wing dynamics of four dragonfly and damselfly species, we draw the following conclusions:

(i) The damping ratio does not significantly vary in different wing regions. Hence, from this aspect, wings can be assumed as underdamped systems with uniform damping ratio.
(ii) The damping ratio of freshly cut wings varies from 0.13 to 0.20. This is considerably higher than that previously thought.
(iii) The wing damping varies between the examined species as well as among fore- and hindwings of the same species.
(iv) We found that the wing hinge significantly influences wing damping. However, in contrast, muscles at the wing base did not influence the wing damping.
(v) Although desiccation significantly decreases the wing damping ratio, rehydration fully restores the damping properties of wings that were dehydrated for 24 h.
(vi) Our results did not show a direct role of hemolymph in the wing damping. However, hemolymph has an indirect influence on the damping properties of the wings by continuously hydrating the wing system.
(vii) The ratio of the flapping frequency and natural frequency of the wings was ~1 for the flier dragonfly species and < 1 for the three examined percher dragonfly and damselfly species.

## Methods

### Ethics

Specimens used in this study were collected with the permission of the Landesamt für Landwirtschaft, Umwelt und ländliche Räume (LLUR) of the state of Schleswig-Holstein, Germany. All the experiments performed in this study comply with the ethical guidelines of Kiel University, and were performed according to the Ordinance on Safety and Health Protection at Workplaces Involving Biological Agents (BioStoffV) launched by the Federal Ministry of Labor and Social Affairs, Germany.

### Animals

In this study, we examined 64 wings from 16 individuals belonging to four different Odonata species. This included three specimens of the dragonfly *A. cyanea* (Fig. 1a, b), four specimens of the dragonfly *S. striolatum* (Fig. 1e, f), four specimens of the damselfly *C. splendens* (Fig. 1c, d) and five specimens of the damselfly *I. elegans* (Fig. 1g, h). The insects were caught in their natural habitats near the river Schwentine (Kiel, Germany) from May to October 2016. They were quickly transported to our lab at Kiel University in air-permeable plastic containers containing humid cotton wools. For transport, the containers were placed in another larger container filled with ice to anesthetize the insects.

### Experimental setup and measurement sites

Prior to each measurement in the laboratory, the insects were anesthetized with CO_2_ and fixed with dental wax to a metal stick (Fig. 2a). The stick was clamped to a micromanipulator, which enabled us to move the insects in any direction when necessary. A paint marker (C. Kreul, Hallerndort, Germany) was used to mark a small point within each of the five measurement sites on the wings (Fig. 2j). Three measurement sites were chosen along with the leading edge spar and two others along the trailing part of the wings. The measurement sites were named as follows: (i) “leading part proximal” (LP) at the proximal part of the leading edge spar, (ii) “nodus” (NOD) near the wing nodus, (iii) “leading part distal” (LD) at the distal part of the leading edge spar near the pterostigma, (iv) “trailing part proximal” (TP) at the proximal part of the trailing margin of the wing, and (v) “trailing part distal” (TD) at the distal area of the trailing margin of the wing.

A laser Doppler vibrometer (Polytec OFV 300 Sensor Head, Polytec GmbH, Waldbronn, Germany) was used to measure the passive return time course of the wings (Fig. 2a). The device was controlled using a Polytec OFV 2100 laser vibrometer controller (Polytec GmbH, Waldbronn, Germany). Prior to the experiment, the laser beam of the device was focused on each marked measurement site. An initial deflection of ~1 cm, which was kept consistent between trials, was applied to the wing tip by the round head of an insect pin connected to a micromanipulator. The oscillations of the wings at each measurement site were recorded versus time via an analogue-digital converter (CED MICRO 1401 mkII, Cambridge Electronic Design Ltd., Cambridge, UK). The data from the measurements were analysed using the software Spike2 (CED Spike2, v.6.18, Cambridge Electronic Design Ltd., Cambridge, UK). The frequency of oscillations and their damping ratio were used as measures to quantify the vibration response of the wing specimens.

### Sample treatments

The return time course measurements were performed on eight groups of differently treated specimens, as follows:

(i) In the first group, wings of anesthetised insects were examined with no particular treatment (Fig. 2b). The results of experiments on the samples in this group are expected to reflect the properties of wings in their natural condition. We call this group the “natural” (nat.) treatment.
(ii) In the second group, the individuals received a magnesium chloride injection (10–25 µl of a 20 mmol/l MgCl_2_ solution) (Fig. 2c) through a very soft cuticle of the tergum, distal to the wing hinge. The MgCl_2_ solution was expected to relax the flight muscles^87^. Therefore, we refer to this as the “relaxed” (rel.) treatment.
(iii) Another group of specimens received a potassium chloride injection (10–25 µl of a 100 mmol/l KCl solution) (Fig. 2d). The solution contracted the flight muscles^88^. Therefore, this treatment is called the “contracted” (con.) treatment.
(iv) In the fourth group, the wings were fixed at their hinge with the body, using a small drop of melted beeswax (Fig. 2e). Since the displacements of the wing base were completely restricted in this group, we call this as the “fixed” (fix.) treatment.
(v) The wings of specimens in the fifth sample treatment group were removed from the insects’ body using sharp scissors. The wings were then sealed and horizontally fixed at their base to the edge of a vertically oriented glass slide with a drop of melted beeswax (Fig. 2f). The glass slide was fixed on a micromanipulator. We performed experiments on these specimens in less than 10 min after they were cut off from the body. This sample treatment is called the “freshly cut” (c-f) treatment.
(vi) Specimens in this group were treated similar to those in the previous group, but after separation from the insect body, they were left for 10 min to allow hemolymph to flow out of the wings (Fig. 2g). The specimens were then tested as the others. This sample treatment is named as the “cut-10 min” (c-10) treatment.
(vii) In this seventh sample treatment, we used wings that were air-dried for >24 h after they were removed from the body (Fig. 2h). This treatment is called the “dry” treatment.
(viii) The specimens used in the previous group were then rehydrated by immersion in distilled water for >24 h, following the widely used protocol described by Klocke & Schmitz^71^ and Aberle et al^89^. (Fig. 2i). After the rehydration, the specimens were subjected to the same type of experiments. We refer to this as the “rehydrated” (rehyd.) treatment group.

To test the influence of certain factors on the oscillatory behaviour of the wings, the results of the experiments in each of the above-mentioned sample treatment groups were compared as follows: The influence of

(i) *flight muscles*, by comparing the results of the “natural” (nat.) treatment group with those of the “relaxed” (rel.) and “contracted” (con.) treatment groups;
(ii) the *wing hinge*, by comparing the results of the “natural” (nat.) treatment group with those of the “fixed” (fix.) group;
(iii) *hemolymph pressure*, by comparing the results of the “fixed” (fix.) treatment group with those of the “freshly cut” (c-f) group;
(iv) the *presence of hemolymph*, by the results of the “freshly cut” (c-f) treatment group with those of the “cut-10 min” (c-10) group;
(v) *desiccation*, by comparing the results of the “freshly cut” (c-f) treatment group with those of the “dry” group;
(vi) *rehydration*, by comparing the results of the “dry” treatment group with those of the “rehydrated” (rehyd.) group.

### Damping ratio and natural frequency

The dimensionless damping ratio, ζ, was used as a measure to describe the oscillatory behaviour of the wings. Taking into account that wings are underdamped systems, which exhibit oscillations, their damping ratio was defined as:^33^

1 $${\rm{\zeta }}=\frac{\delta }{\sqrt{{\left(2\pi \right)}^{2}+{\delta }^{2}}}$$

In the above equation, ${\delta }$ is the logarithmic decrement and represents the rate at which the amplitude of oscillations decreases. It can be measured using the following equation

2 $${\rm{\delta }}=\frac{1}{m}{\rm{ln}}\frac{{x}_{1}}{{x}_{m+1}}$$

where *x*_1_ and *x*_m+1_ are the amplitudes of oscillations corresponding to times *t*_1_ and *t*_m+1_ (m denotes the number of complete cycles between *x*_1_ and *x*_m+1_).

The frequency of oscillations after an initial disturbance, *f*_*d*_, was measured by calculating the number of cycles per second. The natural frequency of specimens, *f*_n_, was calculated using the following equation:^33^

3 $${f}_{n}=\frac{{f}_{d}}{\sqrt{1-{{\rm{\zeta }}}^{2}}}$$

The time-domain logarithmic decrement method applied here is generally used for estimation of the damping ratio of the dominant vibration mode^90^. Given the way the experiment were carried out here, it is likely that we excited this dominant mode; the oscillations of the wing specimens were dominated by large-amplitude bending. Hence, the use of this method in our analysis of the wing damping ratio is justified.

### Statistics and reproducibility

#### Measurement sites

For the results obtained from the different measurement sites the Friedman repeated measures analysis of variance on ranks followed by the Tukey *post hoc* test was used.

#### Fore and hind wings

For intraspecific wing comparisons, we employed the Mann–Whitney rank sum test (Mann–Whitney test). For interspecific comparisons, the Kruskal–Wallis one-way analysis of variance on ranks (Kruskal–Wallis test) followed by the Dunn’s method for multiple comparison was used.

#### Sample treatment

In this category, the one-way ANOVA test (ANOVA) was used for normally distributed data including the Tukey test for multiple comparison. For non-parametric values, the Kruskal–Wallis one-way analysis of variance on ranks (Kruskal–Wallis test) was used followed by Dunn’s method for multiple comparison.

#### Data presentation

Everywhere in the text, *N*, represents the number of data points. All values reported in the text are means ± standard errors. In the box-and-whisker plots, the borders of the boxes indicate the 25th and 75th percentiles, the line within them marks the median, and the whiskers (error bars) define the 5th and 95th percentiles.

### Reporting summary

Further information on research design is available in the Nature Research Reporting Summary linked to this article.

## Supplementary information

Reporting Summary

## Data Availability

The authors declare that the data supporting the findings of this study are available within the paper and its supplementary information files. All source data underlying the graphs and charts can be found at https://figshare.com/s/e677751ab84d1fbf9437.
