# Supplementary material for: The damping and structural properties of dragonfly and damselfly wings during dynamic movement
Source: Commun Biol. 2021 Jun 15;4:737. doi: 10.1038/s42003-021-02263-2 (PMC8206215; doi:10.1038/s42003-021-02263-2)
Supplement: Supplementary file 1 — Reporting Summary [file 42003_2021_2263_MOESM1_ESM.pdf]

nature research

Corresponding author(s): Hamed Rajabi
Last updated by author(s): Apr 16, 2021

Reporting Summary

Nature Research wishes to improve the reproducibility of the work that we publish. This form provides structure for consistency and transparency in reporting. For further information on Nature Research policies, see our Editorial Policies and the Editorial Policy Checklist.

Statistics

For all statistical analyses, confirm that the following items are present in the figure legend, table legend, main text, or Methods section.

n/a	Confirmed	
☐	☒	The exact sample size (*n*) for each experimental group/condition, given as a discrete number and unit of measurement
☐	☒	A statement on whether measurements were taken from distinct samples or whether the same sample was measured repeatedly
☐	☒	The statistical test(s) used AND whether they are one- or two-sided<br>*Only common tests should be described solely by name; describe more complex techniques in the Methods section.*
☒	☐	A description of all covariates tested
☒	☐	A description of any assumptions or corrections, such as tests of normality and adjustment for multiple comparisons
☐	☒	A full description of the statistical parameters including central tendency (e.g. means) or other basic estimates (e.g. regression coefficient) AND variation (e.g. standard deviation) or associated estimates of uncertainty (e.g. confidence intervals)
☐	☒	For null hypothesis testing, the test statistic (e.g. *F*, *t*, *r*) with confidence intervals, effect sizes, degrees of freedom and *P* value noted<br>*Give P values as exact values whenever suitable.*
☒	☐	For Bayesian analysis, information on the choice of priors and Markov chain Monte Carlo settings
☒	☐	For hierarchical and complex designs, identification of the appropriate level for tests and full reporting of outcomes
☒	☐	Estimates of effect sizes (e.g. Cohen's *d*, Pearson's *r*), indicating how they were calculated

Our web collection on statistics for biologists contains articles on many of the points above.

Software and code

Policy information about availability of computer code

Data collection	No software was used.
Data analysis	The software Spike2 (CED Spike2, v.6.18, Cambridge Electronic Design Ltd., Cambridge, UK) to analyze data from passive return time course measurements. The software Sigmaplot (v.12, Systat, San Diego, CA, USA) for the statistical analyses.

For manuscripts utilizing custom algorithms or software that are central to the research but not yet described in published literature, software must be made available to editors and reviewers. We strongly encourage code deposition in a community repository (e.g. GitHub). See the Nature Research guidelines for submitting code & software for further information.

Data

Policy information about availability of data

All manuscripts must include a data availability statement. This statement should provide the following information, where applicable:

- Accession codes, unique identifiers, or web links for publicly available datasets
- A list of figures that have associated raw data
- A description of any restrictions on data availability

All required data are available either in the manuscript or in supplementary materials.

Field-specific reporting

Life sciences study design

All studies must disclose on these points even when the disclosure is negative.

Sample size	In this study, we examined 64 wings from 16 individuals belonging to four different Odonata species. This was the maximum number of specimens that we could collect within the period of the study.
Data exclusions	No data was excluded.
Replication	Each measurement were repeated at least 5 times and all of them were successful.
Randomization	This is not relevant to this study.
Blinding	Investigators were blinded to group allocation.

Reporting for specific materials, systems and methods

We require information from authors about some types of materials, experimental systems and methods used in many studies. Here, indicate whether each material, system or method listed is relevant to your study. If you are not sure if a list item applies to your research, read the appropriate section before selecting a response.

Materials & experimental systems

n/a	Involved in the study	
☒	☐	Antibodies
☒	☐	Eukaryotic cell lines
☒	☐	Palaeontology and archaeology
☐	☒	Animals and other organisms
☒	☐	Human research participants
☒	☐	Clinical data
☒	☐	Dual use research of concern

Methods

n/a	Involved in the study	
☒	☐	ChIP-seq
☒	☐	Flow cytometry
☒	☐	MRI-based neuroimaging

Animals and other organisms

Policy information about studies involving animals; ARRIVE guidelines recommended for reporting animal research

Laboratory animals	This study did not involve lab animals.
Wild animals	We examined 64 wings from 16 individuals belonging to four different Odonata species. This included three specimens of the dragonfly A. cyanea, four specimens of the dragonfly S. striolatum, four specimens of the damselfly C. splendens and five specimens of the damselfly I. elegans. The insects were caught in their natural habitats near the river Schwentine (Kiel, Germany) from May to October 2016. They were quickly transported to our lab at Kiel University in air-permeable plastic containers containing humid cotton wools. For transport, the containers were placed in another larger container filled with ice to anesthetize the insects. Prior to each measurement in the laboratory, the insects were anesthetized with CO_2. After the experiments the insects were euthanized by deep-freezing.
Field-collected samples	This study did not involve samples collected from the field.
Ethics oversight	All the experiments performed in this study comply with the ethical guidelines of Kiel University, and were performed according to the Ordinance on Safety and Health Protection at Workplaces Involving Biological Agents (BioStoffV) launched by Federal Ministry of Labor and Social Affairs, Germany.

Note that full information on the approval of the study protocol must also be provided in the manuscript.
